# Responsive hydrogel microfibers for biomedical engineering

**DOI:** 10.1002/SMMD.20220003

**Published:** 2022-12-27

**Authors:** Jiahui Guo, Zhiqiang Luo, Fengyuan Wang, Hongcheng Gu, Minli Li

**Affiliations:** ^1^ State Key Laboratory of Bioelectronics School of Biological Science and Medical Engineering Southeast University Nanjing China; ^2^ Department of Dermatology Zhongda Hospital Southeast University Nanjing China

**Keywords:** biomedical engineering, microfibers, microfluidics, responsive hydrogel

## Abstract

Responsive hydrogel microfibers can realize multiple controllable changes in shapes or properties under the stimulation of the surrounding environment, and are called as intelligent biomaterials. Recently, these responsive hydrogel microfibers have been proved to possess significant biomedical values, and remarkable progress has been achieved in biomedical engineering applications, including drug delivery, biosensors and clinical therapy, etc. In this review, the latest research progress and application prospects of responsive hydrogel microfibers in biomedical engineering are summarized. We first introduce the common preparation strategies of responsive hydrogel microfibers. Subsequently, the response characteristics and the biomedical applications of these materials are discussed. Finally, the present opportunities and challenges as well as the prospects for future development are critically analyzed.

## Graphical abstract

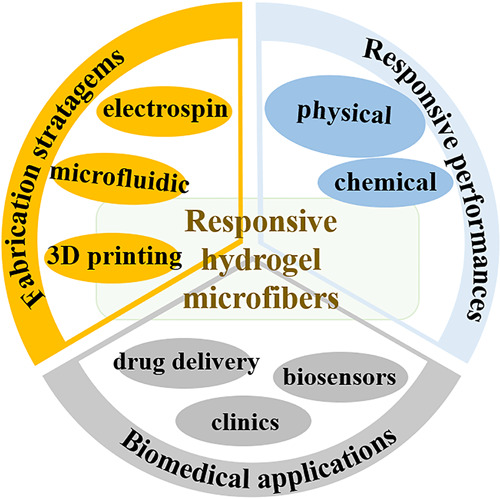

## 1 INTRODUCTION

Hydrogel microfibers are fibrous biomaterials at microscale, with a three‐dimensional network structure, which are generally formed by physical or chemical crosslinking of hydrophilic polymers.^1^, ^2^, ^3^, ^4^, ^5^ Hydrogel microfiber systems have attracted extensive attention and have been demonstrated to systematically combine the advantages of hydrogels (high water content, strong water absorption and retention, excellent biocompatibility, etc.) and the advantages of fibers (variable mechanical properties, diverse structures, weaved properties, etc.).^6^, ^7^, ^8^, ^9^, ^10^, ^11^, ^12^ In recent years, with the rapid development of precision medicine and personalized treatment, the demand for intelligent biomaterials is increasing.^13^, ^14^, ^15^, ^16^, ^17^ As a common intelligent material, the responsive hydrogel system can realize multiple, variable, controllable, and reversible changes in shape or properties under the stimulation of the surrounding environment, so as to meet complex application situations.^18^, ^19^, ^20^, ^21^, ^22^, ^23^, ^24^ A variety of intelligent hydrogels, such as photo‐responsive hydrogels, thermal responsive hydrogels, pH responsive hydrogels, etc., have been developed and shown remarkable biomedical values.^25^, ^26^, ^27^, ^28^, ^29^, ^30^ By combining smart hydrogel systems with fabrication strategies like microfluidic, 3D printing, and electrostatic spinning, the fabrication of responsive hydrogel microfibers could be easily realized, which has achieved significant advances in biomedical engineering applications, including drug delivery, biosensors, clinical treatment, etc., according to the relevant literatures.^31^, ^32^, ^33^, ^34^, ^35^, ^36^

In this review, we provide a comprehensive summarization of the latest relevant research studies on the responsive hydrogel microfibers and their promising applications in biomedical engineering. To begin, we introduce the common fabrication strategy of responsive hydrogel microfibers. Subsequently, attention is focused on their responsive properties, followed by the discussion of the biomedical applications of these materials. Finally, the current opportunities and challenges as well as perspectives on the future developments are critically analyzed.

## 2 FABRICATION STRATEGY

In this section, we would briefly introduce the common fabrication strategies of responsive hydrogel microfibers, including electrospinning technology, microfluidics, and 3D printing (Table 1).

**TABLE 1:** Fabrication strategies of responsive hydrogel microfibers

Fabrication strategies	Characteristics	Advantages	Limits	Examples
Electrospinning	A technique that transforms molten materials into nanoscale ultrathin fibers	Simple, versatile, nanoscale of the resultant fibers	Dependent on the high voltage and the molten state of materials	Responsive fibers with reversible programmable deformation,^45^ fibers with a pH‐sensitive self‐healing ability^46^
Microfluidics	A technology that manipulates fluids in microchannels	Precise control, high integration, diverse interactions of fluids	High technical requirements	Programmable microfibers with reversible deformation,^51^ spindle‐knotted microfibers with photo‐thermal responsiveness,^52^ helical microfibers with magnetic responsiveness^53^
3D printing	A technology that fabricates materials with complex geometric shapes based on computer designs	High accuracy, high cost effectiveness, and custom fiber shapes	Limited choice of materials and time‐consuming	Microfibers with programmed deformation,^57^ microfiber scaffold with photo‐thermal responsiveness^58^

**FIGURE 1:**
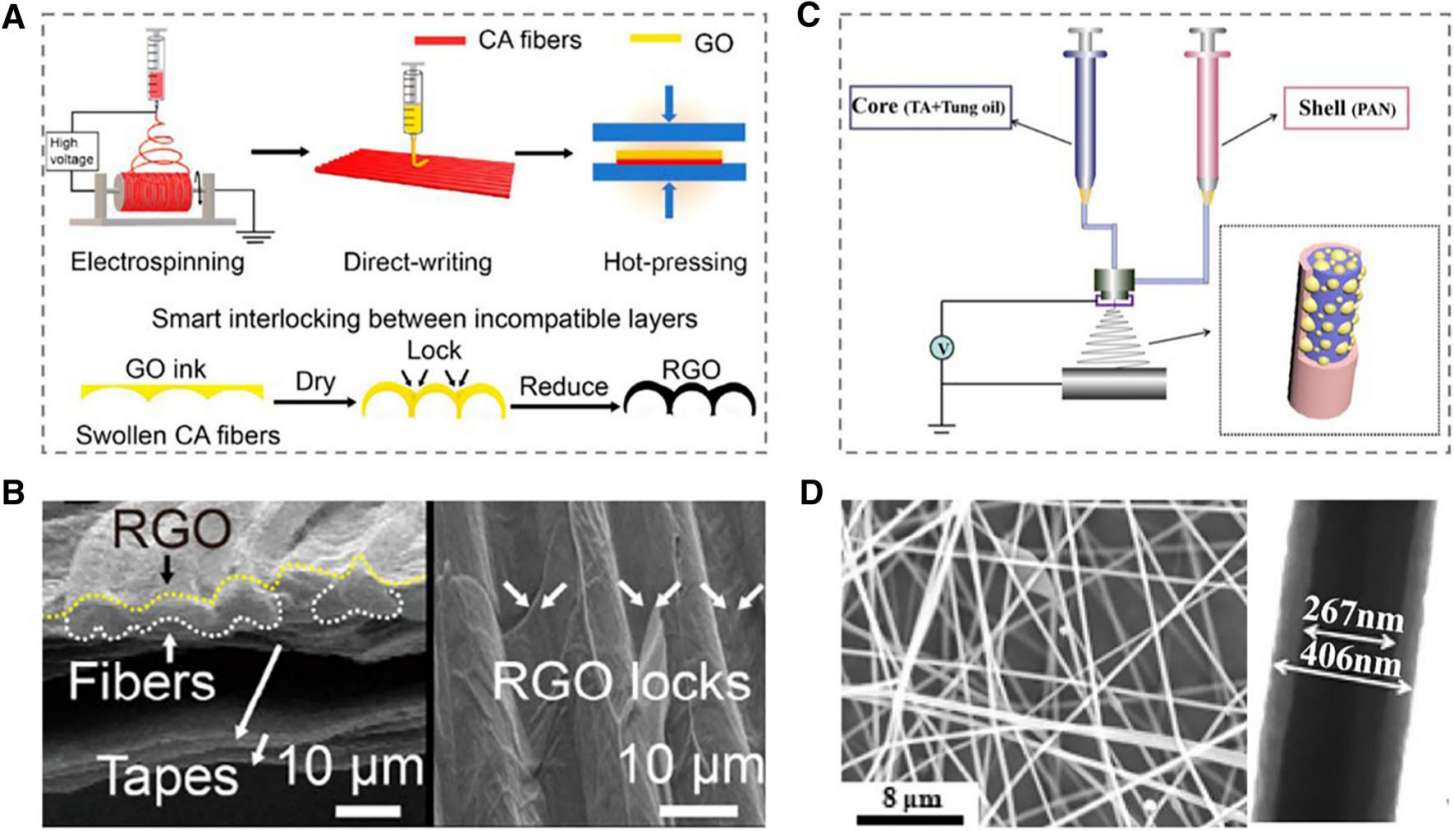
Examples of electrospinning for fabricating responsive hydrogel microfibers. (A) The scheme for the construction process and smart interlocking between layers. (B) SEM images of reduced graphene oxide (RGO)/CA mat. Reproduced with permission.^45^ Copyright 2021, American Chemical Society. (C) Schematic illustration of the fabrication process of the core‐shell electrospun fibers. (D) SEM image and TEM image of the as‐prepared core‐shell electrospun fibers. Reproduced with permission.^46^ Copyright 2021, American Chemical Society.

Firstly, electrospinning technology is a simple but versatile technique to fabricate ultrathin microfibers.^37^, ^38^, ^39^ In recent years, a wide variety of materials, including polymers, composites, and ceramics, have been successfully electrospun into microfibers, mainly in solvent forms and supplemented by melt forms.^40^, ^41^, ^42^, ^43^, ^44^ Dai et al. developed a novel responsive grooved microfiber of cellulose acetate (CA) array by using electrospinning technology (Figure 1A).^45^ Graphene sheets with stimulus response and periodic folds were evenly distributed on the microfibers, giving the fibers rapid and reversible shape changes, multi‐responsiveness, and programmable deformation (Figure 1B). Additionally, Chen's group reported intelligent fibers with pH responsiveness through a coaxial electrospinning method (Figure 1C,D).^46^ With the employment of liquid healing agent as self‐healing materials, the as‐prepared fibers were endowed with a pH‐sensitive self‐healing ability.

**FIGURE 2:**
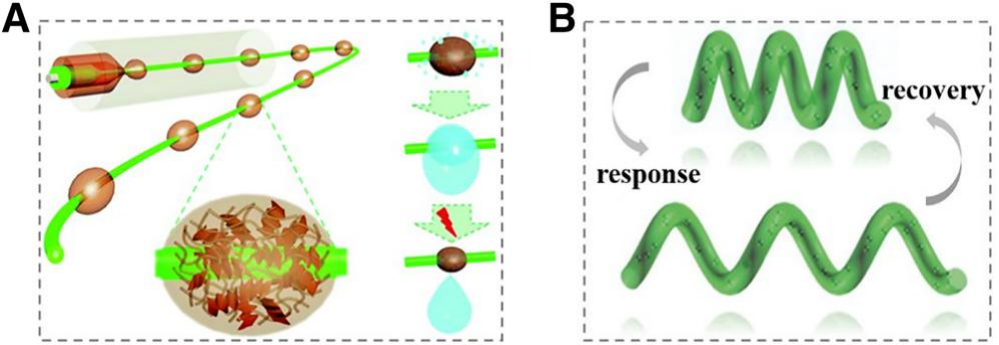
Examples of microfluidic technologies for fabricating responsive hydrogel microfibers. (A) Concept of the spindle‐knotted microfiber with photo‐thermal responsiveness for water collection. Reproduced with permission.^52^ Copyright 2017, Royal Society of Chemistry. (B) Scheme for magnetically responsive deformation of helical microfiber. Reproduced with permission.^53^ Copyright 2017, John Wiley and Sons.

Microfluidics technology, which is characterized by precise manipulation of fluids at the micro scale, has been exhibiting considerable promise in fabricating functional materials.^37^, ^47^, ^48^, ^49^, ^50^ Onoe et al. proposed a microfiber‐shaped programmable material that can flexibly respond to external stimuli and have explored their potential applications in the fields of bionics and soft robotics.^51^ In addition, Zhao's group reported spindle‐knotted graphene oxide (GO) microfibers with photothermal responsiveness for water collection through microfluidic technology combined with spinning and emulsification procedures (Figure 2A).^52^ Furthermore, helical microfibers could be produced through microfluidic technology, which could be imparted with magnetic responsiveness when magnetic nanoparticles were integrated.^53^ To be specific, when the magnet was close to the hydrogel helical microfibers, the spiral pitch would increase obviously, while it would recover to the original state when the magnets left (Figure 2B).

**FIGURE 3:**
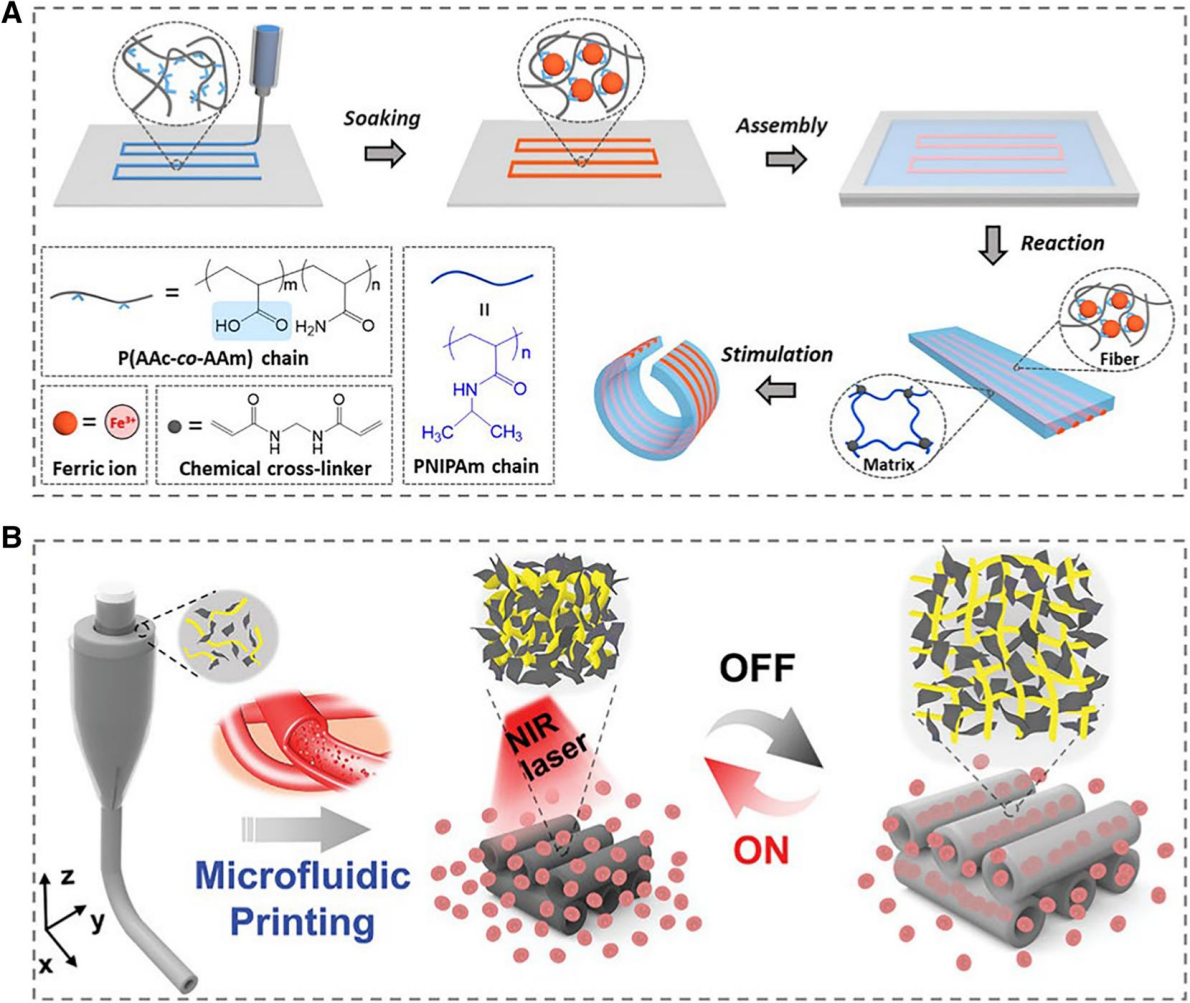
Examples of 3D printing for fabricating responsive hydrogel microfibers. (A) Schematic illustration for the fabrication procedure of the composite hydrogel. Reproduced with permission.^57^ Copyright 2020, American Chemical Society. (B) Schematic diagram of the BP scaffold fabrication from microfluidic 3D printing, and its responsive ability under near‐infrared (NIR) irradiation. Reproduced with permission.^58^ Copyright 2021, John Wiley and Sons.

3D printing, also called as additive manufacturing (AM), is rapidly developed for instant machining and manufacturing, which has been expected to fabricate materials with complex geometric shapes based on computer designs.^54^, ^55^, ^56^ Zheng et al. embedded hydrogel microfibers into a hydrogel matrix by using 3D printing technology (Figure 3A).^57^ Based on the different responses of microfibers and gel matrix to an external stimulus, the programmed deformation control of composite gel was realized, which was valuable for biomimetic fields, biomedical engineering, flexible electronics, etc. Similarly, by using microfluidic 3D printing technology, Zhao's group successfully prepared black phosphorus (BP) composite microfiber scaffold with photothermal responsiveness, whose value in promoting vascular formation and bone regeneration has been verified (Figure 3B).^58^

## 3 RESPONSE PERFORMANCES

Under the environmental stimulation, hydrogels would undergo multiple, variable, controllable, and reversible changes in the manner of space or time according to their own characteristics or the unique properties of the incorporated materials. These external stimuli include exogenous stimuli, such as magnetic field, electric field, temperature, etc., and endogenous stimuli, such as pH, enzyme concentration, etc. In this section, we will focus on the response performances of diverse responsive hydrogel microfibers.

**FIGURE 4:**
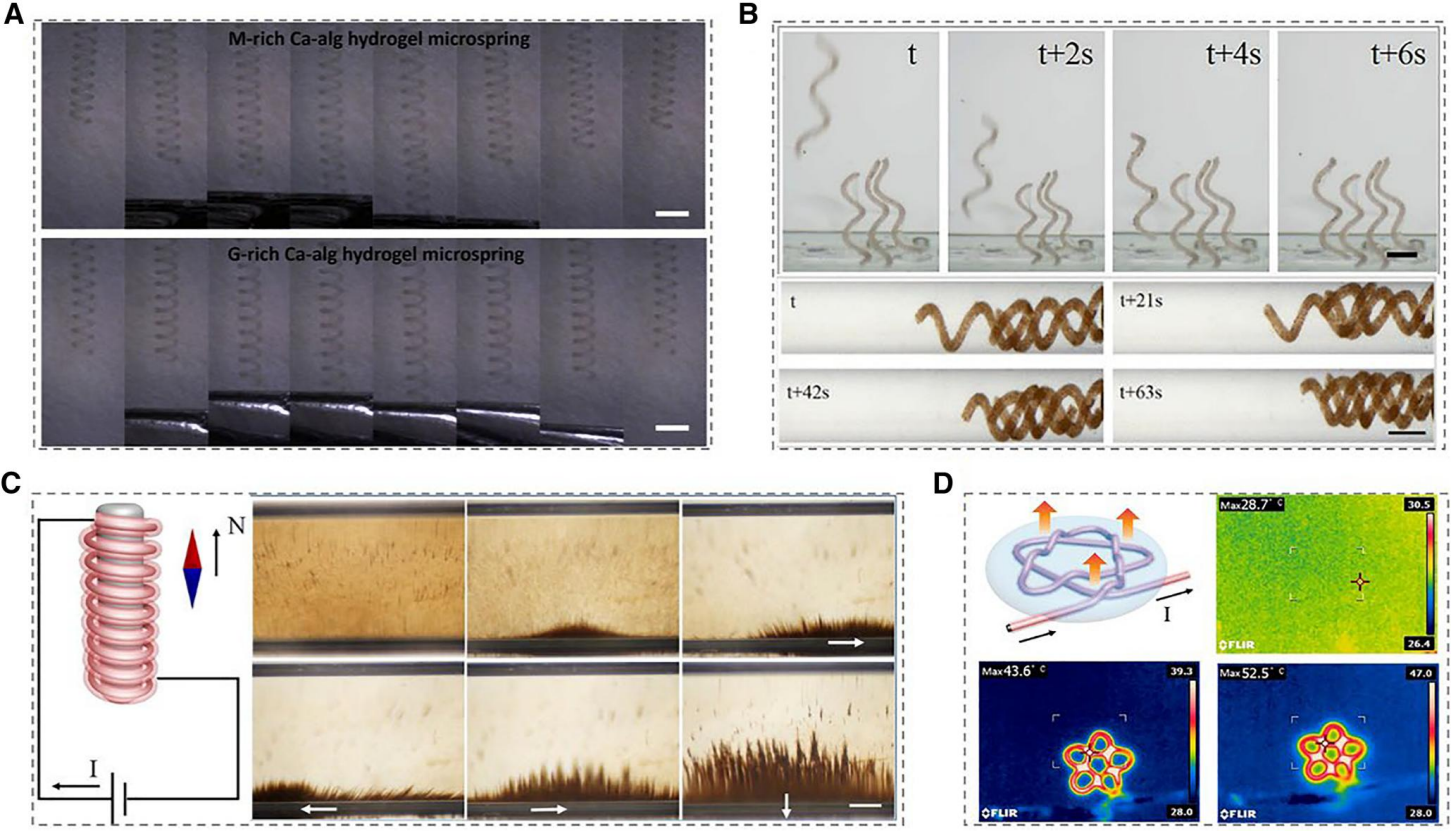
Hydrogels with magnetic and electrical response. (A) Microscopic images of hydrogel microfibers under diverse magnetic forces. Scale bars are 1.5 mm. Reproduced with permission.^65^ Copyright 2020, Elsevier. (B) Time‐lapse sequences of helical micromotors forming a plane array and triple‐helical structure. Scale bars are 300 and 150 μm, respectively. Reproduced with permission.^67^ Copyright 2020, American Chemical Society. (C) Characterization of the electromagnet conversion of microfibers. Scale bar is 250 μm. (D) Electrothermal conversion of the as‐prepared microfibers. Reproduced with permission.^73^ Copyright 2020, Elsevier.

Magnetic phenomenon exists widely and is well‐known as one of the earliest physical phenomena in nature.^59^, ^60^ Even in the human body, specific tissues and organs would produce weak magnetic fields that accompany life. Additionally, with the in‐depth research on magnetic responsive hydrogels, it is found that these hydrogels have the advantages of easy manipulation, wide application range, and good biocompatibility, thus having been widely used in biomedical engineering.^61^, ^62^, ^63^, ^64^ Mi et al. generated coil microfibers with magnetic features by mixing magnetic Fe_3_O_4_ nanoparticles with the sodium alginate solution.^65^ The response performance of the resultant helical microfibers to magnetic stimulation was then investigated, which proved their potential values as responsive micro‐springs, nano‐swimmers, or actuators (Figure 4A). Similarly, Shang et al. realized the wireless rotation of the helical microfibers with magnetically responsive characteristics under the control of six degrees of freedom electromagnetic system with different frequencies.^66^ Furthermore, Zhao's group presents micromotors of helical microfibers driven by magnetic field as dynamic cell microcarriers.^67^ Thanks to the special helical structure of the hydrogel microfiber and the magnetic responsiveness of the introduced magnetic nanoparticles, these hydrogel microfibers could perform parallel movement and rotated locomotion under the action of the magnetic field, which were further assembled into different geometric structures, including planar arrays and tubular structures (Figure 4B).

In addition to magnetic field, electricity also plays an irreplaceable role in human's daily life. The electrostimulation‐responsive materials exhibit the advantages of quick response and accurate adjustment, and some examples of electrically stimulated drug release have been demonstrated.^68^, ^69^, ^70^ Xiong et al. developed one conductive hydrogel microfiber that could respond to electrical stimulation for the controlled release of water‐soluble drug molecules.^71^ In addition, hydrogel of gelatin and poly(3,4‐ethylenedioxythiophene): poly(styrene sulfonate) (PEDOT:PSS) could realize a sustained drug release, which was stimulated by electrical ion varying from 0 to 1.5 V.^72^ Besides, it is worth exploring the behavior of the responsive microfibers to convert electricity to magnetism or heat under electrical stimulation.^73^, ^74^ Zhao's team proved the feasibility of electromagnetic conversion by demonstrating that ferrofluid motion could be realized when electrically stimulated microfibers were coiled on an iron rod (Figure 4C).^73^ Additionally, thanks to the electrothermal effect, microfibers have been proven as thermal management devices under electric stimulation (Figure 4D).

**FIGURE 5:**
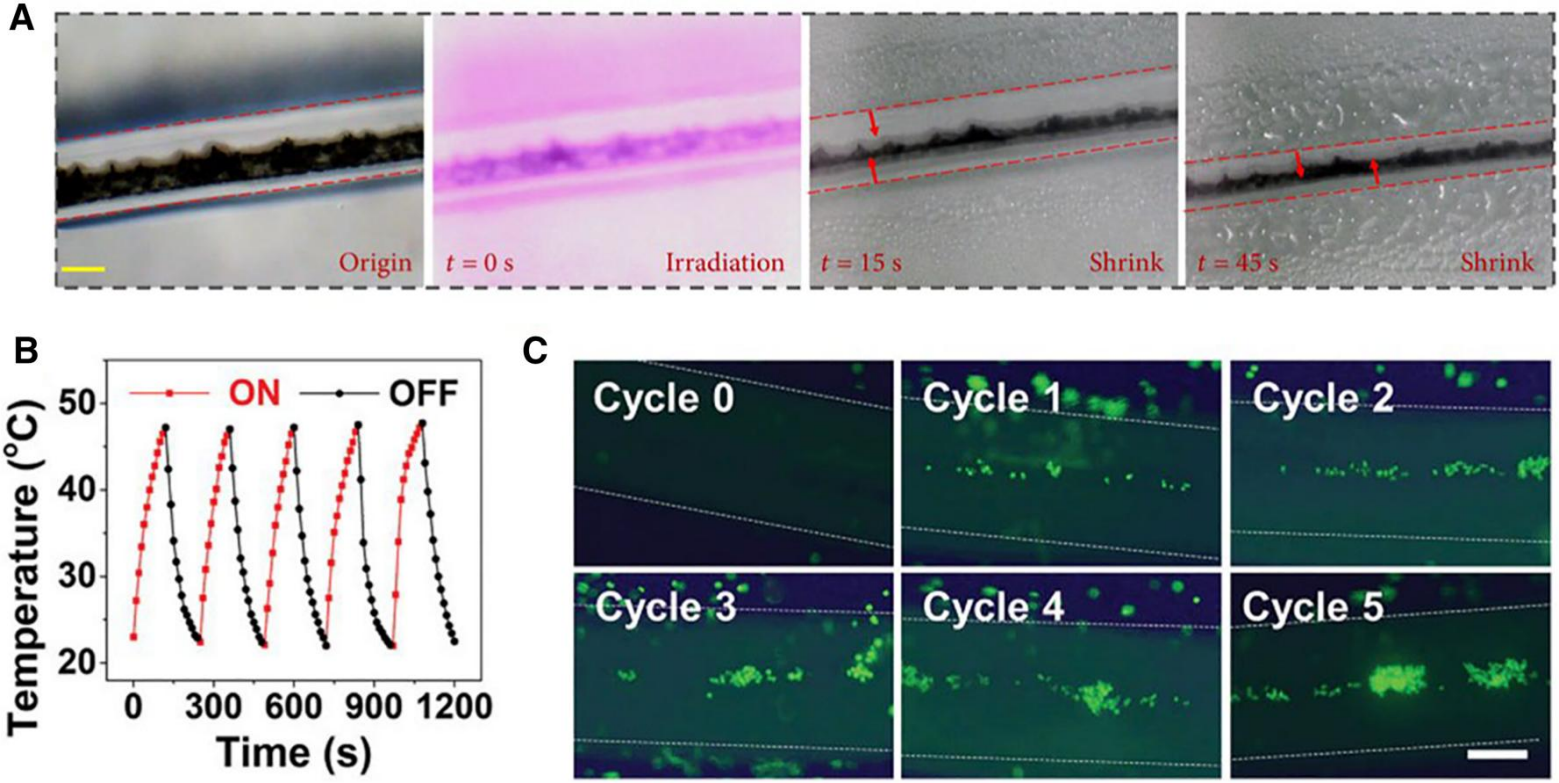
Typical examples of temperature‐responsive hydrogel microfibers. (A) Images of the microfiber shrinkage with the increasing temperature. Scale bar is 210 μm. Reproduced under terms of the CC‐BY license.^4^ Copyright 2021, The Authors, published by American Association for the Advancement of Science. (B) Temperature variation of BP composite microfiber scaffold over five cycles under NIR irradiation. (C) Fluorescent images of cells in microchannels after 5 NIR cycles. Scale bar is 300 μm. Reproduced with permission.^58^ Copyright 2021, John Wiley and Sons.

Temperature is another common physical stimulus. Temperature‐responsive materials triggering temperature changes in the physiological range have attracted much attention for their potential values in biomedical applications.^75^, ^76^, ^77^, ^78^ The microfiber‐shaped programmable material proposed by Onoe et al. was found to exhibit localized and repeated deformations in the SR poly(N‐Isopropylacrylamide‐co‐acrylic acid) (pNIPAAm‐co‐AAc)/Ca‐Alginate region under cycled temperature stimulation (heated to 40°C and cooled to 20°C).^51^ Temperature stimulation could also be achieved by NIR irradiation based on the addition of photosensitive response elements.^79^, ^80^ The microfibers with MXene core and PNIPAM shell were induced to shrink when the overall temperature of the fiber was increased under NIR laser irradiation (Figure 5A).^4^ In addition to MXene, the combination of BP nanosheets and poly(N‐Isopropylacrylamide) (PNIPAM) also showed excellent photothermally responsive properties (Figure 5B).^58^ The scaffold of the composite materials has the characteristics of controllable expansion and contraction under the temperature stimulation of NIR irradiation, which could further promote the enrichment of cells in the scaffold channel under repeated stimulation (Figure 5C). Hydrogel materials added with GO or reduced graphene oxide (RGO) have also been proved with photothermal responsive properties, which were applied to enrich water and catalyze reactions.^45^, ^52^, ^81^

**FIGURE 6:**
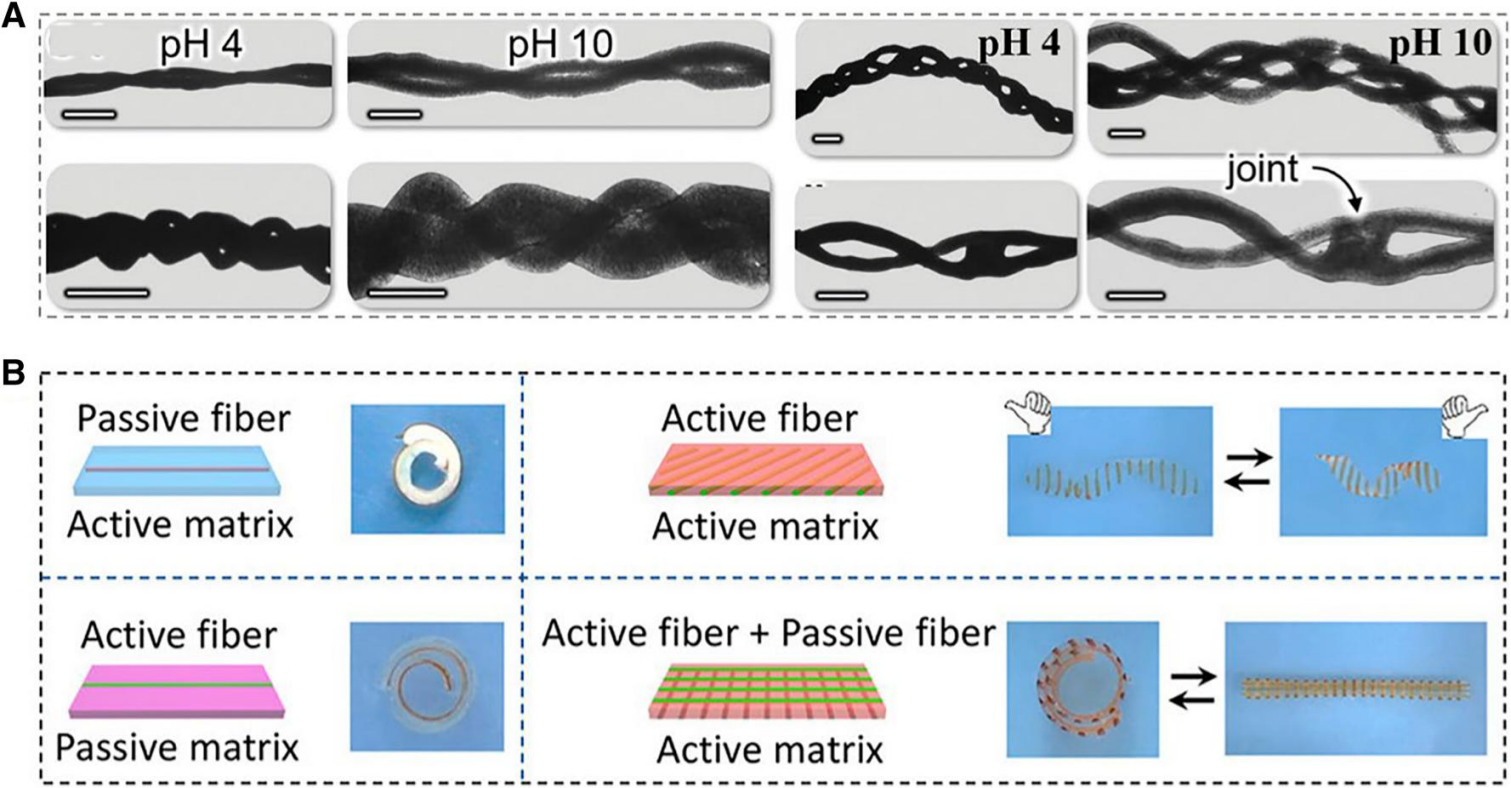
Examples of pH‐responsive hydrogels. (A) SNP/pAA hydrogel fiber microropes during pH increasing. Reproduced with permission.^84^ Copyright 2022, The Authors, published by John Wiley and Sons. (B) Multiple shape transformations of the composite hydrogels. Reproduced with permission.^57^ Copyright 2020, American Chemical Society.

Since physiological activities of humans are always accompanied by pH changes, pH‐responsive hydrogels, which could be greatly sensitive to external environmental stimuli, are recognized as a class of advanced intelligent biomaterials.^82^, ^83^, ^84^, ^85^ The silica nanoparticle (SNP)/polyacrylic acid (pAA) composite microfibers prepared by Haase et al. were proved to be pH‐responsive.^84^ During the increase of pH values from 4 to 10, the spiral twisted microfibers expanded rapidly in volume with enhanced mechanical flexibility (Figure 6A). In addition, polyacrylonitrile fibers based on tannic acid (TA) and tung oil (TO) have been shown to be pH‐sensitive.^46^ Under the stimulation at different pH values, TA and TO exhibited a pH‐sensitive healing effect on the cracking coating, which showed the potential for long‐term corrosion resistance. Furthermore, Zheng et al. utilized the difference between the pH responsiveness of microfibers and gel substrates to integrate active and passive responses, eventually achieving programmed deformation of materials (Figure 6B).^57^

Apart from the responsive hydrogels described in detail above, many other responsive hydrogels, such as enzyme‐responsive, humidity‐responsive, electrochemistry‐responsive, ultrasound‐responsive, etc., have been utilized to construct responsive hydrogel microfibers, thus playing an irreplaceable role in the biomedical field, such as catalysis, drug delivery, sensing, and tissue engineering.^86^, ^87^, ^88^, ^89^, ^90^

## 4 APPLICATIONS

### 4.1 Drug delivery

**FIGURE 7:**
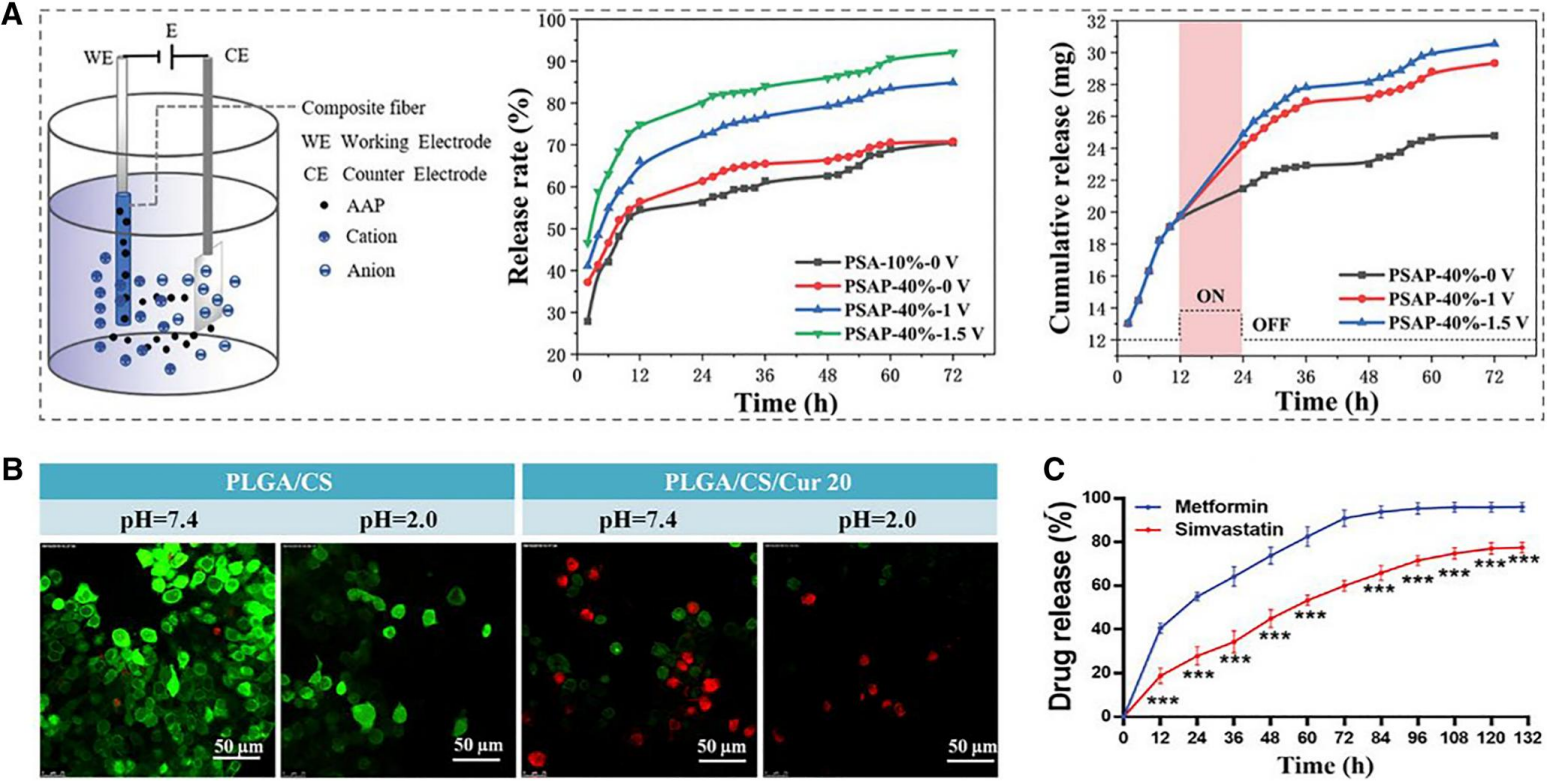
Application of responsive hydrogel microfibers for drug delivery. (A) Release profile of AAP from microfibers under electrical stimulation. Reproduced with permission.^71^ Copyright 2022, John Wiley and Sons. (B) Cell activity of different fiber groups under different pH values. Reproduced with permission.^96^ Copyright 2021, Elsevier. (C) Drug release from fibers with hierarchical structure over the time. Reproduced with permission.^98^ Copyright 2021, American Chemical Society.

Controlled release of drugs can reduce the toxic and side effects of drugs on humans to a certain extent. And delivering drug to specific target sites could improve the therapeutic effect to reduce the administration times of drugs, eventually achieving the effect of relieving pain of patients.^91^ Responsive hydrogels are of great values in drug delivery and controlled release due to their dynamic response to the environmental stimuli.^92^, ^93^ Generally, in response to exogenous stimuli (such as temperature, magnetic field, etc.) or endogenous stimuli (such as pH, enzyme concentration, etc.), responsive hydrogels would undergo protonation, hydrolysis, cleavage, or (super) molecular conformational changes, thus promoting the controlled release of the encapsulated drugs. For instance, the fabricated conductive microfibers, which were composed of biomaterials (polyvinyl pyrrolidone and sodium alginate), conductive materials (PEDOT: PSS), and model drug acetaminophen (AAP), were found with obviously enhanced drug release under electrical stimulation.^71^ Especially, controlled drug release could be achieved by controlling microfiber conductivity and the employed electrical fields or by applying continuous/intermittent electrical stimulus (Figure 7A). The proposed microfiber was expected to be a drug delivery patch for wearable smart healthcare systems, relieving patients' pain on demand. Similarly, quercetin (Q), known as a natural flavonoid, was added to fibers consisted of polylactic acid (PLA) and GO.^94^ And it was found that, under appropriate electrical stimulation, Q was released much faster than traditional drug release, ensuring complete drug release.

Notably, cancer cells would secrete acidic metabolites due to anaerobic glycolysis, causing that the pH value of tumor tissue and its surroundings tend to below 6.8, compared with that (pH 7.4) for normal tissues.^95^ Therefore, the development of local drug delivery systems with pH responsiveness for the treatment of cancer deserves a great deal of attention. Wang et al. investigated the release of curcumin (Cur) from polylactic glycolic acid/chitosan/Cur (PLGA/CS/Cur) fibers at different pH values.^96^ It has been demonstrated that the release amount of Cur under acidic conditions is much higher than that under normal conditions, showing higher anticancer activity (Figure 7B). CS/pectin/hydroxypropyl‐γ‐cyclodextrin (HPγCD)/Cur fibers achieved up to 89% Cur loading efficiency.^97^ In addition to high swelling properties, the prepared fibers also showed pH‐responsive release capacity of Cur in media of pH 5.4 and 7.4. Besides, novel enzyme‐ and relative humidity (RH)‐ responsive antibacterial fibers were developed.^98^ Specifically, when enzymes secreted by microorganisms triggered a fiber response, the fibers were able to release free antimicrobial active ingredients (AIs), while AIs in the form of cyclodextrin‐inclusion complexes (CD‐ICs) were released at higher relative humidity (95% RH). The controlled release of metformin and simvastatin responding to temperature was achieved in a novel fibrous membrane composite system. It was demonstrated from the release curve that drugs released explosively in the first few hours, while they tended to release in a slow and sustained manner for the rest of the time (Figure 7C). Generally, controlled drug delivery could realize the applications of administering a specific dose at a specific time and site, which is of great value in many fields, such as disease treatment.

### 4.2 Biosensors

**FIGURE 8:**
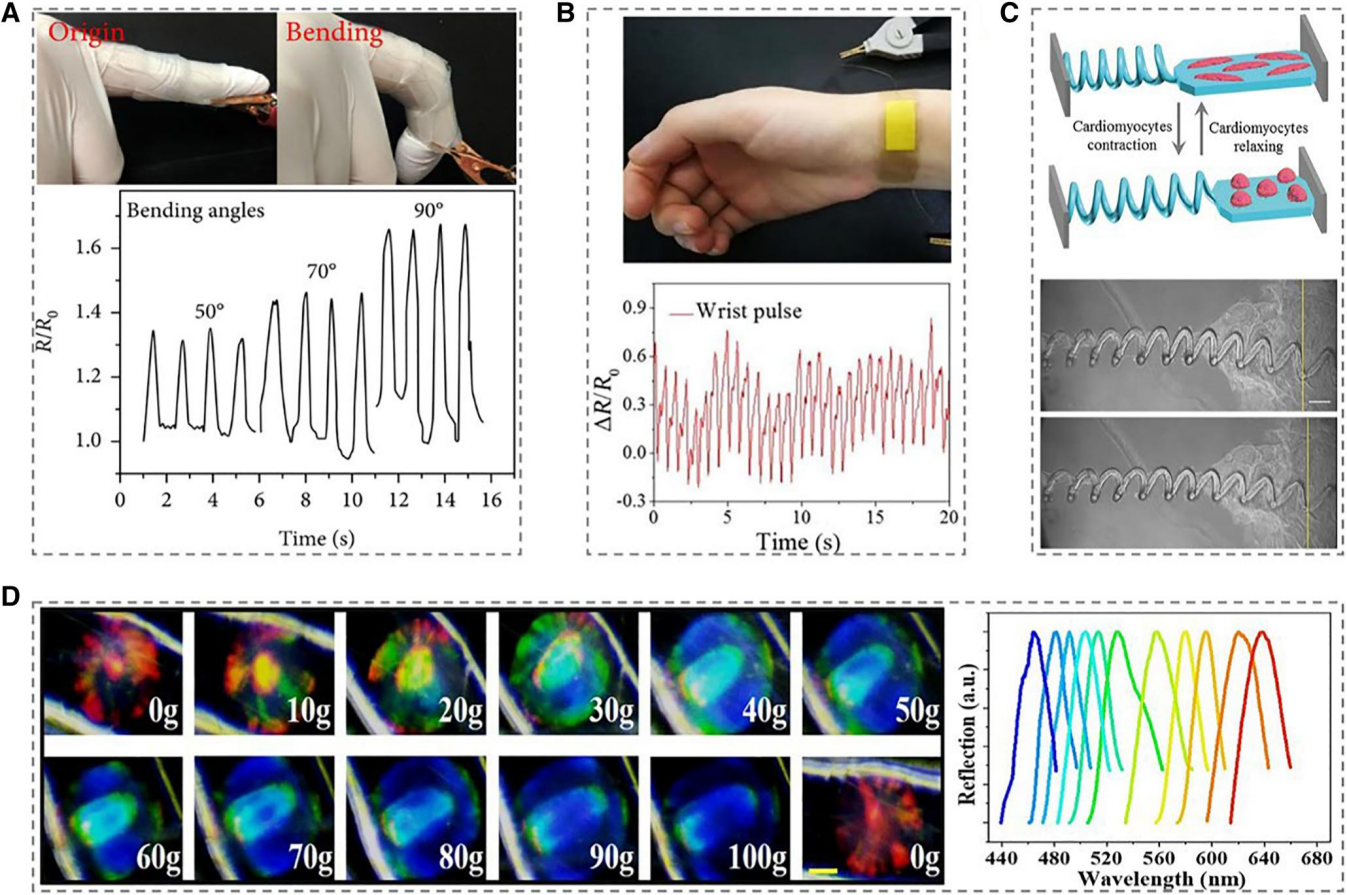
Applications of responsive hydrogel microfibers for biosensors. (A) Conductivity response to finger bending in real time. Reproduced under terms of the CC‐BY license.^4^ Copyright 2021, The Authors, published by American Association for the Advancement of Science. (B) Flexible membranes with LM‐microfibers for wrist pulse sensing. Reproduced with permission.^73^ Copyright 2020, Elsevier. (C) Schematic illustration and microscopy images of helical microfibers for sensing cardiomyocytes. Scale bar is 150 μm. Reproduced with permission.^53^ Copyright 2017, John Wiley and Sons. (D) Real‐time microscopy photographs and the corresponding spectral reflection of the red microspheres during the compression process. Scale bar is 100 μm. Reproduced with permission.^101^ Copyright 2022, Springer Nature.

Biosensor is a kind of monitoring device, which can transform environmental information, such as force, temperature, humidity, etc., into electrical or optical signals that can be easily monitored. They have been reported to be applied in diverse fields, including flexible electronics, wound monitoring, human–computer interaction, and some others.^99^, ^100^ For example, the prepared conductive microfibers encapsulated with MXene could be used as strain sensors to monitor human activities in real time.^4^ The bending movement of the finger was transferred into electrical signals (resistance), and the bending degree of finger could be inferred from the magnitude of the change in its relative electrical resistance, which was also applicable to monitor wrist and elbow movement (Figure 8A). In addition, microfibers integrated with liquid metals (LMs) also had important practical applications in wearable electronics.^73^ The membrane system with the fabricated microfibers could then be used to monitor the wrist pulse, which is an important physiological signal of systolic and diastolic blood pressure and heart rate (Figure 8B). To be specific, the elastic membrane system embedded with LM‐integrated microfibers was firstly attached to the inside of bandage and then adhered to the wrist. Periodic arterial pressure from the wrist caused real‐time changes in the relative resistance of the fibers, enabling dynamic monitoring of pulse rate.

Apart from monitoring the human activity from a macro perspective, responsive microfibers can also be used to monitor mechanical sensing of cardiac muscle cells.^53^ When the helical microfibers were connected to the hydrogel membrane implanted with cardiomyocytes, these microfibers could be observed extending and contracting with the beating of cardiomyocytes on the hydrogel membrane, and their transformation cycle frequency was linked with the cardiomyocytes beating frequency (Figure 8C). In addition to mechanical and electrical signals, responsive microfibers could also convert external stimuli into optical signals.^101^ Under mechanical actions, the microfibers implanted with discontinuous red structural color microspheres showed a color gradient from red to green and then to blue (Figure 8D). The applied force was corresponding to the blue shift of the microsphere spectrum, which confirmed its ability as a tactile sensor with spatial identification. Generally, with the rapid development of modern technology, biosensors could be combined with artificial intelligence to realize better human–computer interaction in a dynamic environment, eventually promoting the development of biosensors in a more versatile direction.^102^

### 4.3 Disease treatment on animal models

**FIGURE 9:**
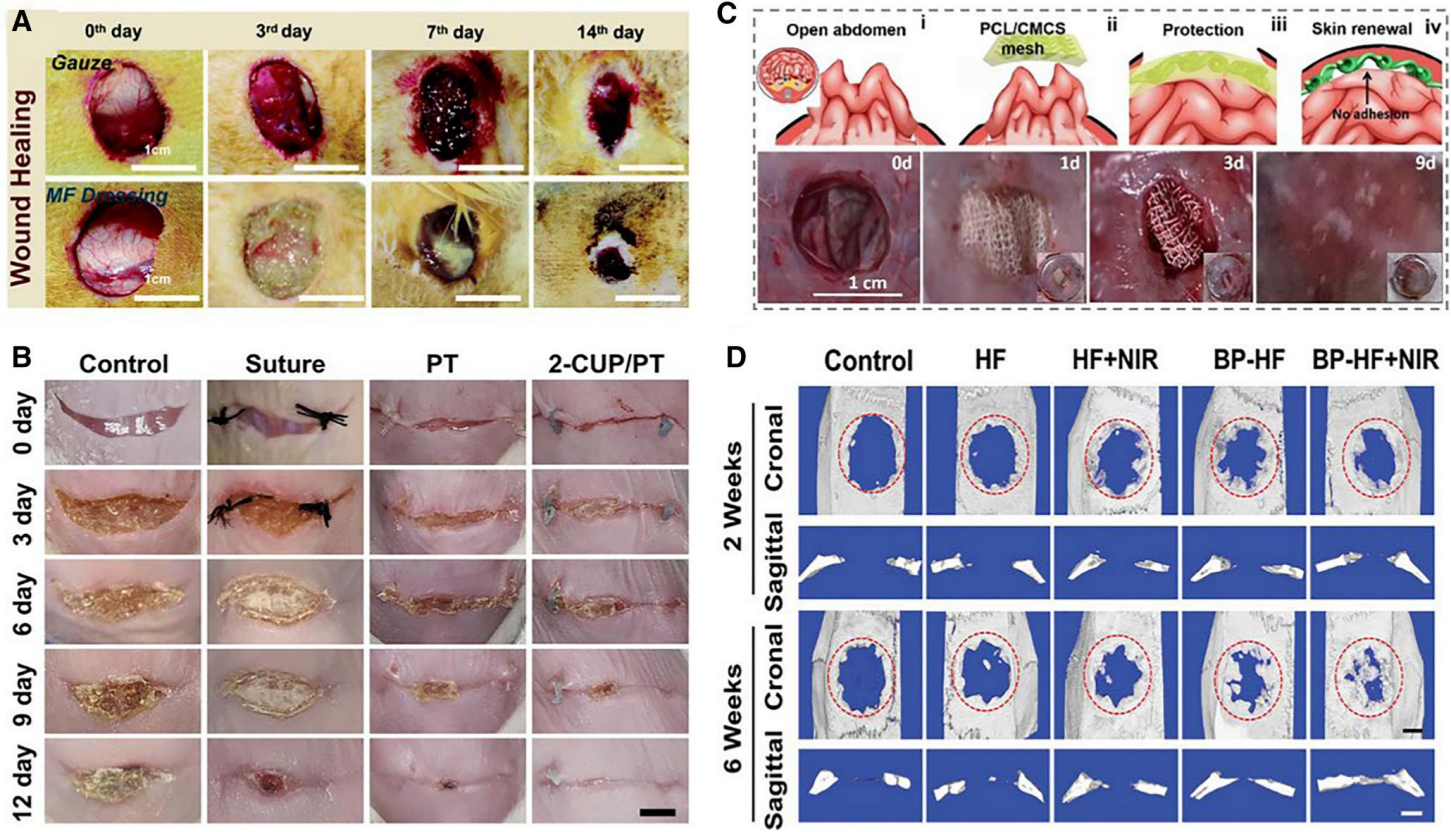
Application of responsive hydrogel microfibers for clinical treatment. (A) Photos of the wound healing process by using different dressings. Reproduced with permission.^105^ Copyright 2022, Royal Society of Chemistry. (B) Wound images with different treatments. Reproduced with permission.^106^ Copyright 2021, Elsevier. (C) Representative photographs of PCL/CMCS mesh as an artificial abdominal wall. Reproduced with permission.^108^ Copyright 2021, John Wiley and Sons. (D) Characterization of bone regeneration under various treatments. Reproduced with permission.^58^ Copyright 2021, John Wiley and Sons.

With the rapid development of social economy and the improvement of residents' living standards, people's demand for the comfort of disease treatment is increasing year by year. Diabetic foot wound, as a common disease with high prevalence, high recurrence probability, and long duration, is in urgent need of an effective treatment.^103^, ^104^ Multifunctional medical dressings have been developed through 3D printing by Yu et al., which showed excellent biocompatibility, controlled drug release property, remarkable antibacterial, and anti‐inflammatory functions.^105^ After a 14‐day animal study in type 2 diabetic rats, the multifunctional dressing was found to effectively reduce inflammatory responses and promote the period of diabetic wound healing (Figure 9A). Apart from dressings, sutures are also widely used in surgical procedures for wound treatment. In order to deal with some complex and severe wound closure, new biodegradable intelligent sutures have been developed.^106^, ^107^ A light‐responsive biomaterial was developed and exhibited excellent functions of shape memory, continuous release of ionic drugs, and light‐triggered self‐bonding ability. And then it was employed as smart suture, showing enhanced wound closure and speeded wound healing properties (Figure 9B).^106^ Additionally, to prevent incisional hernia formation and promote wound healing without adhesion, a flexible, stretchable, biocompatible helical hydrogel microfiber was prepared and applied to artificial abdominal skin (Figure 9C).^108^

In addition to wound treatment, bone injury is another common globally disease that lacks effective treatments, which is often caused by traffic accidents or bone diseases. Since tissue engineered scaffolds have been widely used in the treatment of bone defects, hydrogel microfiber scaffolds with photothermal response have been developed for bone regeneration^58^. The microfiber scaffold showed excellent capacities to promote cell proliferation and osteogenic differentiation in vitro. Besides, after implantation in vivo, the scaffold channel promoted the formation of blood vessels, thus effectively accelerating healing processes of bone defects (Figure 9D). Besides, responsive hydrogel microfibers have also been applied in other organ regeneration studies. For instance, Gotoh et al. constructed hydrogel microfibers to culture human pluripotent stem cells (hPSCs) for lung regeneration^12^. With the assistance of responsive microfibers, expanded hPSC‐derived lung progenitors (hLPs) were successfully implanted into mice lungs, paving the way for cell‐based treatment of end‐stage lung diseases.

## 5 CONCLUSION AND OUTLOOKS

Responsive hydrogel microfibers could sense subtle changes of physical and chemical quantities from the surrounding environment, including pH, temperature, pressure, etc., and accordingly change their physical or chemical properties, which have been widely investigated and applied in biomedical engineering fields. In this review, we provide a comprehensive summarization of the recent relevant research progress of the responsive hydrogel microfibers and their applications prospects in biomedical engineering. The fabrication strategy of responsive hydrogel microfibers in common use is introduced firstly, followed by the introduction of their responsive properties. Subsequently, the biomedical applications of these responsive hydrogel microfibers in drug delivery, sensing, and clinical therapy are introduced. With the rapid development of precision medicine and personalized treatment, the development of intelligent responsive hydrogel microfibers is still worth anticipated.

Although remarkable progress has been made in this field, there remain challenges among the design, preparation, and practical application of materials. Based on these, the following important issues need to be addressed to achieve the wider application of responsive hydrogel microfibers in the biomedical fields. First of all, hydrogel materials need to be endowed with excellent biocompatibility, including their inherent properties such as degradability and adaptability in vivo, which are of great significance for biomedical applications. In addition, the material structures are also crucial for biomedical applications, which could receive inspirations from unique structures in nature. Besides, the influence of the fabrication strategy on the responsiveness and biocompatibility of hydrogel microfibers should also be thoroughly analyzed and evaluated to ensure their effectiveness. Last but not least, the complexity of real biomedical applications, including physiological environment and complex human body structure, should be fully analyzed to achieve a more comprehensive application. Therefore, we do believe that with further joint efforts of scientists of different study backgrounds, responsive hydrogel microfibers would be pushed forward to become more applicable and reliable for biomedical engineering fields.

## AUTHOR CONTRIBUTIONS

Fengyuan Wang and Hongcheng Gu conceived the idea; Jiahui Guo wrote the manuscript and edited the figure; Zhiqiang Luo revised the manuscript; Minli Li supervised the manuscript.

## CONFLICTS OF INTEREST

The authors declare no competing financial interests.
